# Supplementary figures and images for: DNA methylation insulates genic regions from CTCF loops near nuclear speckles
Source: eLife. 2025 Sep 3;13:RP102930. doi: 10.7554/eLife.102930 (PMC12408068; doi:10.7554/eLife.102930)

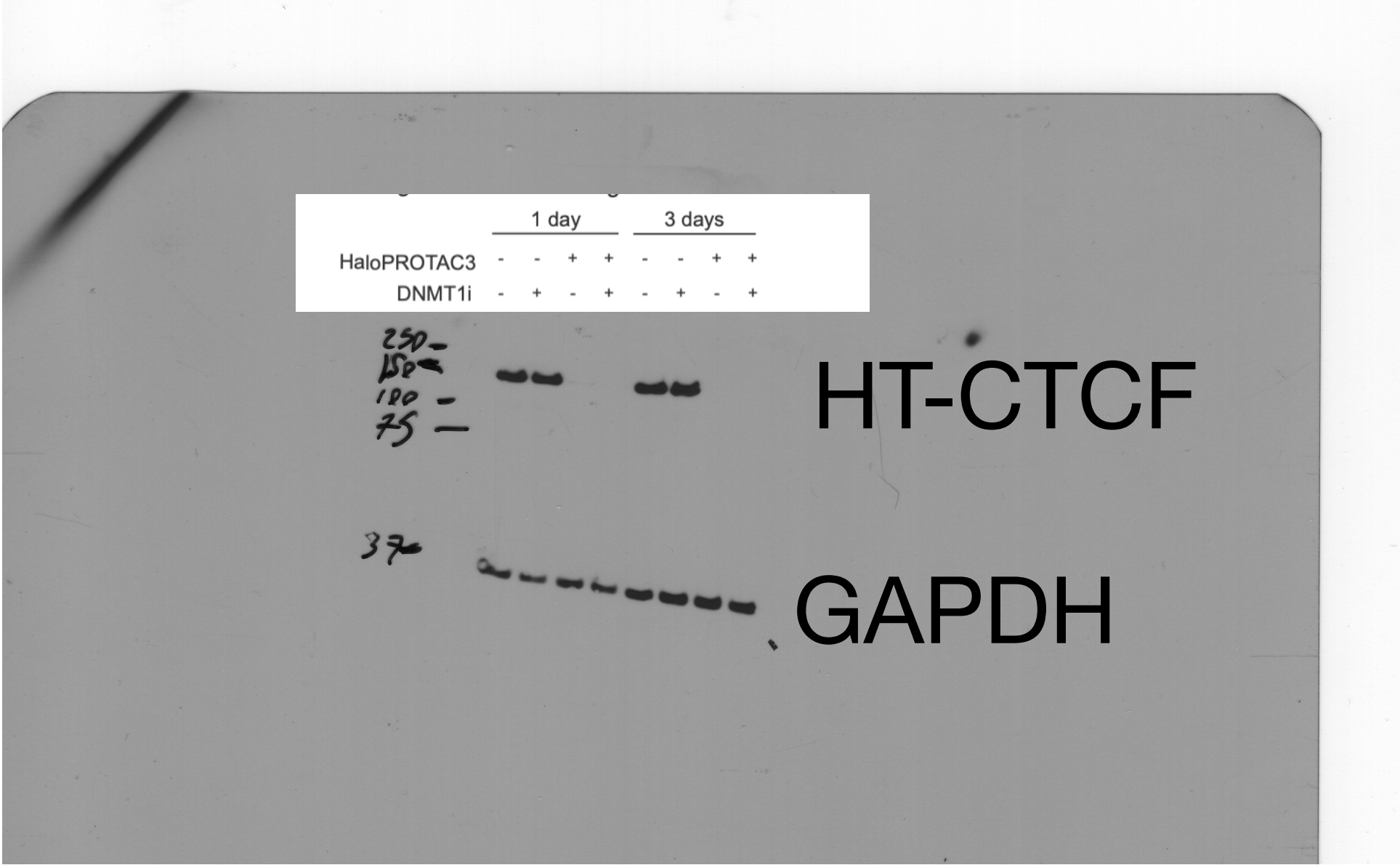

Supplement: Figure 3—source data 1. [file elife-102930-fig3-data1.pdf]

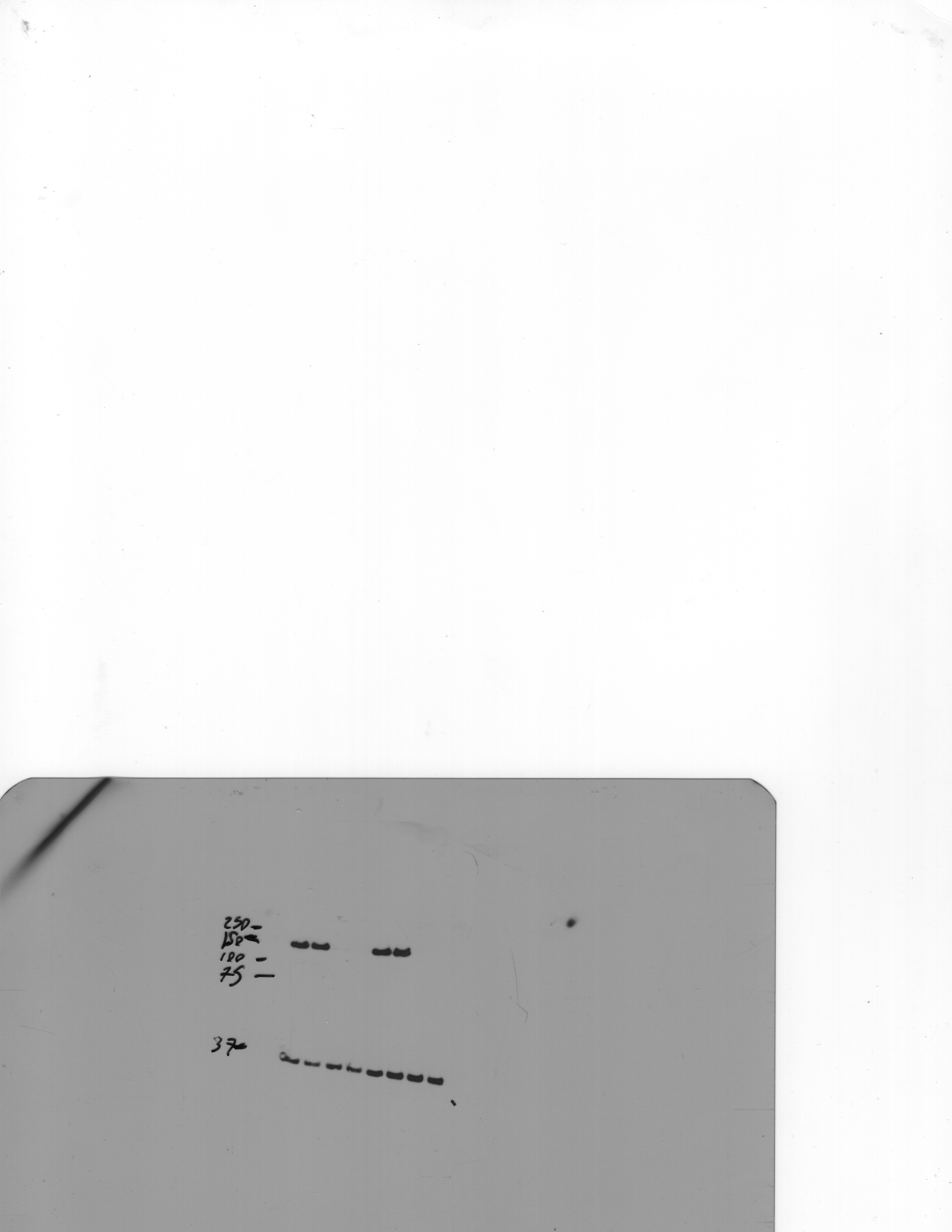

Supplement: Figure 3—source data 2. [file elife-102930-fig3-data2.zip › Figure 3 - Source Data 2.jpg]

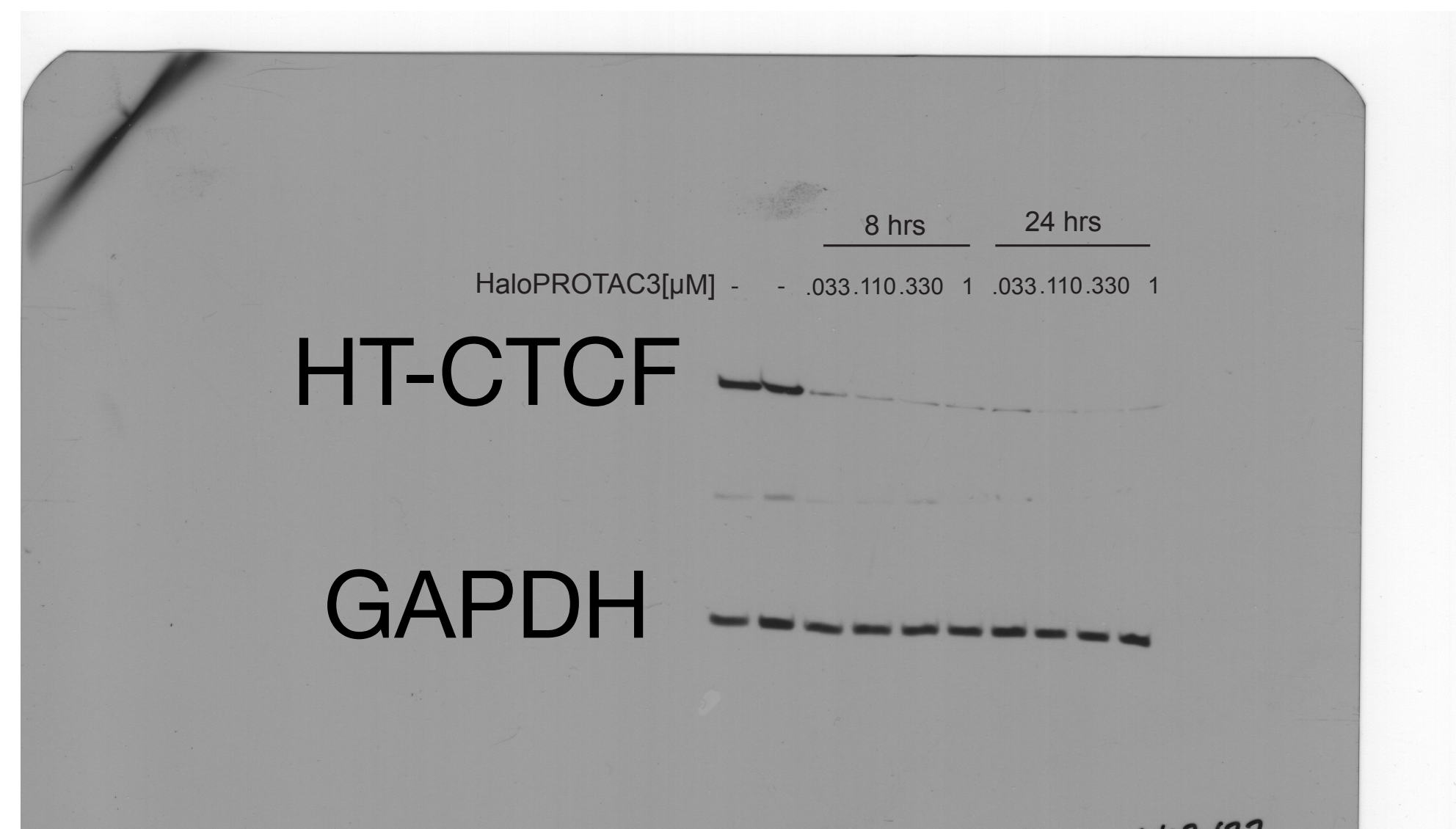

Supplement: Figure 3—figure supplement 1—source data 1. [file elife-102930-fig3-figsupp1-data1.pdf]

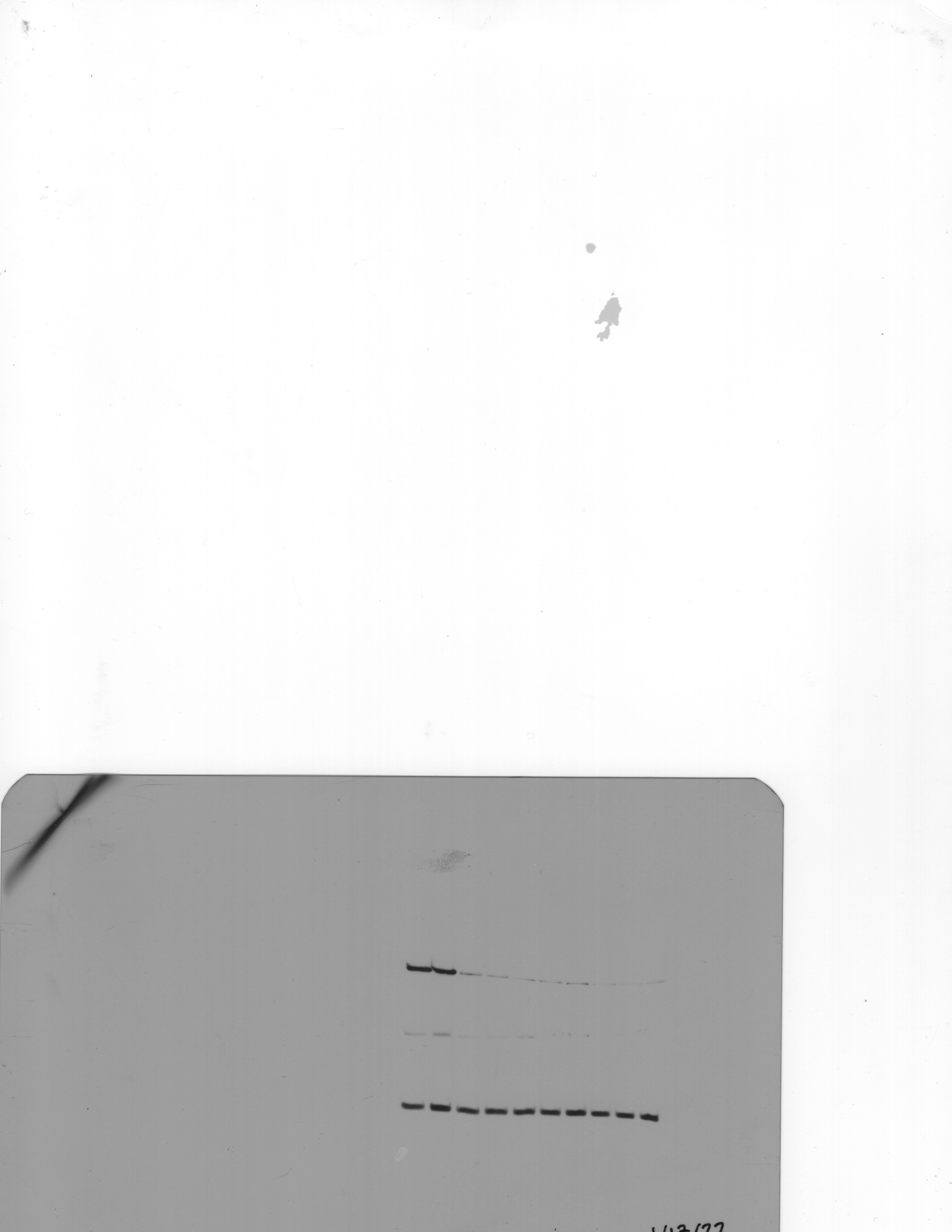

Supplement: Figure 3—figure supplement 1—source data 2. [file elife-102930-fig3-figsupp1-data2.zip › Figure 3 - Figure Supplement 1 - Source Data 2.tiff]

# SR330\_V4

|         | Knockin |   |   |   |   |   | Wt |
|---------|---------|---|---|---|---|---|----|
| Dtag-13 | -       | + | - | + | - | + | -  |

SON

250

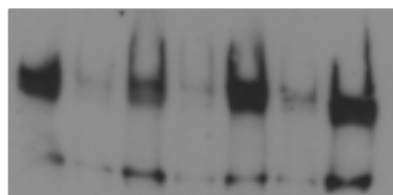

SRRM2

250

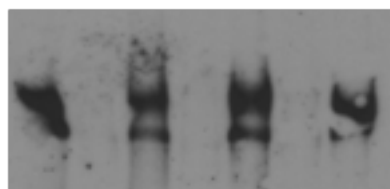

vinculin

150 —  
100 —

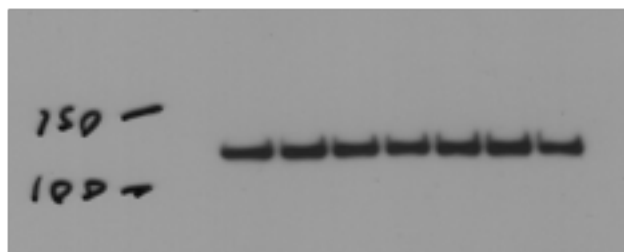

Supplement: Figure 4—figure supplement 1—source data 1. [file elife-102930-fig4-figsupp1-data1.pdf]
